# A dataset of recorded electricity outages by United States county 2014–2022

**DOI:** 10.1038/s41597-024-03095-5

**Published:** 2024-03-05

**Authors:** Christa Brelsford, Sarah Tennille, Aaron Myers, Supriya Chinthavali, Varisara Tansakul, Matthew Denman, Mark Coletti, Joshua Grant, Sangkeun Lee, Karl Allen, Evelyn Johnson, Jonathan Huihui, Alec Hamaker, Scott Newby, Kyle Medlen, Dakotah Maguire, Chelsey Dunivan Stahl, Jessica Moehl, Daniel Redmon, Jibonananda Sanyal, Budhendra Bhaduri

**Affiliations:** 1 Oak Ridge National Laboratory, Oak Ridge, TN 37830 USA; 2 Los Alamos National Laboratory, Los Alamos, NM 87544 USA; 3 Cadre5, Knoxville, TN 37932 USA; 4 Steampunk, Inc, McLean, VA 22102 USA; 5 National Renewable Energy Laboratory, Golden, CO 80401 USA

**Keywords:** Power distribution, Energy access, Energy security

## Abstract

In this Data Descriptor, we present county-level electricity outage estimates at 15-minute intervals from 2014 to 2022. By 2022 92% of customers in the 50 US States, Washington DC, and Puerto Rico are represented. These data have been produced by the **Environment for Analysis of Geo-Located Energy Information (EAGLE-I**^**TM**^**)**, a geographic information system and data visualization platform created at Oak Ridge National Laboratory to map the population experiencing electricity outages every 15 minutes at the county level. Although these data do not cover every US customer, they represent the most comprehensive outage information ever compiled for the United States. The rate of coverage increases through time between 2014 and 2022. We present a quantitative Data Quality Index for these data for the years 2018–2022 to demonstrate temporal changes in customer coverage rates by FEMA region and indicators of data collection gaps or other errors.

## Background & Summary

The 2003 Northeast blackout was an acute failure of the US-Canada electric power system. The outage left over 50 million people without power for up to four days and was initially sparked by electrical faults occurring when high voltage power lines sagged into tree branches. These initial faults cascaded into the largest power outage in North America, in a context in which inadequate situational awareness was described as the second of four major causes of the event^1^. It is believed that the event’s cascading consequences could have been averted or mitigated if utilities had visibility and situational awareness of their surrounding utility partners. It took several hours to estimate that the event left over 50 million people without electricity. This event motivated the U.S. Department of Energy (DOE) to create a wide-area situational awareness and visualization capability^2^ which came to be known as **Environment for Analysis of Geo-Located Energy Information (EAGLE-I**^**TM**^**)**.

**Fig. 1:**
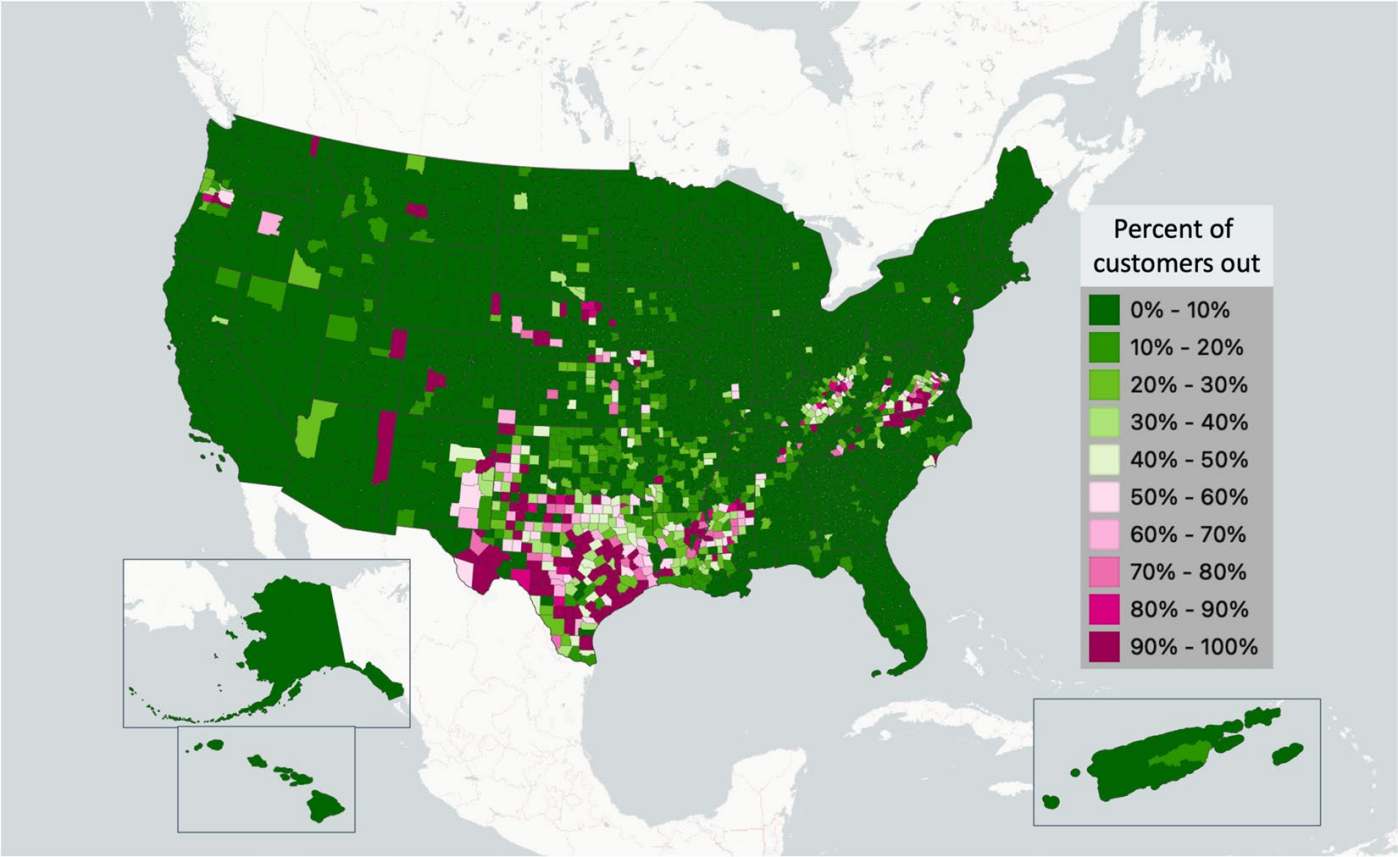
County level EAGLE-I power outage data during winter storm Uri. For each county, we show the maximum percentage of customers that simultaneously experienced an outage for all of the 15-minute collection intervals between February 12 and 19, 2021. These outages did not occur simultaneously during this week, this map represents the circumstances that were “as bad as it ever was” in the focal period.

**EAGLE-I** (https://eagle-i.doe.gov) is a geographic information system, data visualization, and situational awareness platform created to monitor electric utility customer outages from data gathered from public sources^3^. EAGLE-I reports electricity service outages at 15-minute intervals for 3,044 out of 3,226 US counties and county equivalents by 2022, starting from 2,152 in 2014. EAGLE-I is a critical mission support function for the U.S. Department of Energy’s Office of Cybersecurity, Energy Security, and Emergency Response (CESER) and provides authoritative information on energy impacts to other federal and coordinating agencies including the Executive Office of the President, the National Security Council, the Department of Homeland Security (DHS), the Federal Emergency Management Agency (FEMA), the Federal Energy Regulatory Commission (FERC), and the North American Electric Reliability Corporation (NERC). Federal, regional, state, and local planners, utilities and emergency responders use EAGLE-I for situational awareness and decision making, especially while addressing major disasters such as hurricane Ian (2022), winter storm Uri (2021), and the Camp Fire (2018). Figure 1 shows maximum outages for winter storm Uri. In addition to providing critical situational awareness, this platform is building a long-term record of electricity disruptions. Like records of past weather events, records of climate driven infrastructure service disruptions are important for understanding characteristics of the coupled infrastructure-climate system in order to better anticipate the consequences of future climate events^4–9^.

In this data descriptor, we present a complete set of validated historic EAGLE-I records, including eight years of county level power outage information from 2014 to 2022 at 15-minute intervals, and county-level estimates of electricity customer population. We also present five years of Data Quality Statistics for the 10 FEMA Regions (2018–2022). Although these data do not cover all US customers, they represent the most comprehensive outage information ever compiled for the United States. The coverage rate increases through time between 2014 and 2022. Precise data quality statistics became available in 2018. In 2018, outage data was collected from 339 individual electrical utilities, which account for 137 million (86%) electric utility customers. By 2022, our outage data covers 456 individual electrical utilities, which account for 146 million (92%) electric utility customers. These data include all US states, the District of Columbia, and some territories. Puerto Rico and the Virgin Islands are included, while Guam, the American Samoa, and the Northern Mariana Islands are not included. The remaining 8% of customers belong to utilities which do not report outage information publicly in near-real time in a format that is currently accessible to EAGLE-I parsers. These are most typically small, rural, municipal utilities which lack robust information technology infrastructure.

Historic electricity outages can be inferred from a range of different sources. Researchers have collaborated with electric utilities to access high resolution outage data for limited scope and duration^10^. Satellite imagery such as Night Time Lights has been used in disaster response circumstances for assessing outage intensity and spatial characteristics^5,11^. Social media posts^6,12^ and automatic pings by internet enabled devices have also been used to assess outages^13^, typically for specific adverse events or in limited contexts.

The most significant large scale data sources on electricity outages results from the DOE Office of Electricity’s required reporting on Electricity Disturbances, through form OE417^14–17^. These data are compiled from federally required reporting, provide comprehensive coverage for large outages, and also include some information on the cause of the disturbance. OE417 data are reported at the county or state level, and so do not always disaggregate outages across counties. The OE417 data are comprehensive for outages affecting more than 50,000 customers or 300 MW. There are three key dimensions through with EAGLE-I data are more precise than the OE417 data. EAGLE-I data systematically include smaller outages. EAGLE-I data are processes to provide county scale outage estimates. EAGLE-I data report customers out in 15 minute increments, while OE417 data are event based, reporting only a start and end time for the event. Fundamentally, the OE417 data and EAGLE-I data are complementary: EAGLE-I’s underlying data source is information made available to the public in real-time including both small and large outages, and has processed to create uniform spatial scale estimates, while OE417’s basis is mandated reports about specific events.

The EAGLE-I data may be useful for assessing the consequences of adverse weather events on electricity infrastructure, determining geographic, environmental, heterogeneity in electricity service vulnerability, or validating power systems models. For example, these data could be used to quantify differences in grid resilience across different balancing authorities, across states, or in different climate zones. These data could be used to explore changes in grid resilience or robustness over time, by different seasons, or as associated with macroeconomic conditions. They could be used to explore the macroeconomic consequences of experiencing long term or repeated outages.

These data represent a large share of customers, and a large share of outages, but there are gaps. Outages with a duration less than 15 minutes are not reliably captured. Outages within small, rural, or independent electric utilities are not as reliably captured as outages within larger utilities or electricity conglomerates. Over long time periods, or large spatial scales, changes in utility population and inconsistencies in data quality may influence relative data accuracy and precision. Our associated Data Quality Index provides an indicator of the times and locations through which we estimate the outage data is most accurate and complete.

## Methods

There are an estimated 3,000 electric utilities in the United States, but their sizes vary widely. In 2021, Duke Energy was the largest electric utility in the United States and served just over 8 million customers, while half of the utilities in the US serve less than 10,000 customers, with a collective customer population of 2.8 million customers (1.5% of total)^18^. Many utilities collect and publicize real-time outage information for their customers and service area on institutional websites. For identified utilities which do so, we collect, parse, aggregate, and validate these self-reported outages directly from utility websites.

There are no requirements on the format of real-time outage reports, although the Outage Data Initiative Nationwide (ODIN)^19^ encourages utilities to follow either the CIM IEC 61968-3 or MultiSpeak standard. Additionally, website technology and uptime are dynamic. As a result, maintaining active parsers for each included utility requires substantial continuous maintenance and has required significant investment in checks and validation. We have achieved widespread coverage of electric outages by focusing initially on large utilities and subsequently on geographic areas with sparse coverage. We use a wide range of web parsing techniques to systematically collect near real-time outage information from several hundred of the United States’ large electric utilities and utility conglomerates, which are entities that report electric outages from a collection of electric utilities.

The EAGLE-I outage data represents 80% to 90% of utility customers nationally, with higher coverage rates later in our dataset. The coverage extent increased as additional parsing capabilities were developed and as data sharing agreements with other response agencies and utilities were formalized. If an electric utility is not included in EAGLE-I monitoring, that utility is omitted from EAGLE-I data calculations. If a county has no covered electric customers because all utilities (one or more) associated with that county are not monitored by EAGLE-I, then that county is omitted from EAGLE-I reports and data calculations. In 2014, 1,072 counties had no outage data collected, while in 2022, only 182 counties had no outage data collected, out of 3,222 total counties and county equivalents.

### Defining & estimating total customers per county

It is important to recognize that our estimate of utility customers cannot be directly translated to population. Utilities define “customers” in a range of different ways, most typically the electric meter, a building, or a facility. In residential locations, a customer might be a household, while in a commercial location, a customer may be a business or a facility.

These parsed data report the estimated number of customers experiencing an outage, but the number of customers by geographical region is not always provided. To present a geographically consistent measure of outage severity, we also need to generate an estimate of total customers for each utility for each county in the EAGLE-I monitoring process.

We estimate the total number of customers by county using county population, customer totals from each utility, and the coverage area of each utility. County population is derived from LandScan USA (https://landscan.ornl.gov/) using the average of daytime and nighttime population^20,21^, information on utility customer totals is collected from the Energy Information Administration’s form 861 (EIA-861) (https://www.eia.gov/electricity/data/eia861/), and Electric Retail Service Territories geospatial coverage data is drawn from Homeland Infrastructure Foundation-Level Data (HIFLD) (https://hifld-geoplatform.opendata.arcgis.com/).

The total count of electric utility customers is proportionally allocated to daytime and nighttime population totals within the utility service area, based on the utility’s service area’s ratio of customers to population.

When *C* is total customers in the service area, *P* is total population in the service area, *p*_*i*_ is population in county *i* within the service area, *c*_*i*,_ customers in county *i* is calculated as

$${c}_{i}={p}_{i}\ast \frac{C}{P}$$

### Collecting outage data

Our customer outage data collection process [or extract, translate, and load (ETL)] monitors public electric utility customer outage websites every 15 minutes and updates the growing dataset. The outage information posted by utilities is highly variable, encompassing a range of spatial resolutions, ancillary data, and data production methods. Sometimes, the total number of customers served is provided by the utility, in other cases it is based upon our modelled estimate of customers in each county^22^. Outage websites feed automatically from the utility’s outage management system, with manual updating used in cases when a utility’s outage management system fails. We additionally employ a suite of strategies in near real time to address errors and gaps in reported outages.

Our overall data production process aims to synthesize, systematize, and adjust for variance in utility outage reporting statistics to the greatest extent possible. However, these data are fundamentally dependent upon what is reported publicly by electrical utilities. The wide variety in reporting mechanisms between utilities is carried forward to into our aggregated data, and also introduces unavoidable temporal inconsistencies into our data. In general, automated data reporting extent and reliability improved between 2014 and 2022.

The 15-minute return period was selected to balance data recency and precision with computational cost. Fifteen minutes is also the duration threshold for federal electricity disturbance reporting. This return period mean that outages with a duration of less than 15 minutes are only captured if they happen to be represented at the moment when the web-parsers extract data from the utility’s website: 10 minute outages have a 2/3 chance of being represented, while 5 minute outages have a 1/3 chance of being represented.

### Web-parsing strategy

Utilities use many different mapping platforms to report outages. The data formats we encounter most often are JSON, ebill, and IFactor. The EAGLE-I data team has created dozens of parser types and individual scripts that collect reported outage information. Table 1 shows the number of utilities covered by each parser type. Once a collection pathway has been implemented for a utility, it is included in our collection run. Each collection run starts at the 15-, 30-, 45-, and 60-minute mark of each hour. Each run takes less than 5 minutes to parse all outage maps.

**Table 1:** Distribution of utilities whose data is collected through each parser type used in EAGLE-I data collections of January 2023.

Utility Count	Data Format
125	JSON_Parsers
95	eBill
60	Custom Parser
40	IFactor
33	XML_Parsers
24	MilSoft
24	OutageEntry
18	HTML_Parsers
11	json_byfeatures
7	State_Coverage
5	PolyIFactor

### Parser maintenance

The maintenance of the EAGLE-I data collection pipeline is managed by a team which monitors error reports daily. The EAGLE-I Utility Error Count report is produced daily and details the number and type of errors experienced by each utility. Bugs are created for these errors and two releases per week go out to the ETL pipeline to ensure the utility parser collects accurate outage information in a timely manner. This is the process that often identifies when a utility has updated their map URL, a new county is added to a utility’s coverage, or a map has changed the format of the outage data reported.

Each week, a data team member will do a series of additional, broader checks to identify other potential issues that will affect the quality of the outage data. First, the Utility Repeated Outages report is checked to see if a utility has reported the same number of outages for more than four days. If this occurs, the utility is manually checked by the data team to ensure (1) the number collected matches the outage map and (2) the outage map is still the best source of information for the utility. Often, we find a utility creates a new outage map and leaves the old URL functional with outdated outage information – which appears as repeated outages. Second, the Utility Data Quality report is used to identify utilities that have not reported an outage within the past week. The parser is run manually, and outage numbers are visually compared to the outage map for that utility. If there is a mismatch, a bug is created for the data team to get this parser reporting correct outages quickly. If, based on the best available map for that utility, it appears no outages have occurred, this utility is added to a task that weekly monitors utilities without recent outages to ensure everything is working as expected.

Conglomerate feeds are a method to reduce required EAGLE-I maintenance. Certain states, such as Illinois and Iowa, have electricity cooperatives which collect outage information from multiple utilities and present all outages in one conglomerate map. This decreases the maintenance needed for utility parsers in this state, as we may need to only update and monitor one website instead of thirty. For example, the Iowa Association of Electric Cooperatives reports outages from 39 different distribution utilities throughout the state of Iowa, which we parse through a single map interface.

### Synthesizing to consistent geographies

After parsing the raw data, we implement a geographic normalization procedure to create county level records. Aggregating outages to the county level presents utility outage data at a consistent, standardized resolution. While most outage maps provide information at the county level, we often see outage data provided at resolutions of point, polygon, and zip code. Table 2 reports the raw spatial resolution of reported information for the utilities in our collection.

**Table 2:** Distribution of Spatial Resolutions.

Utility Count	Resolution
210	County
191	Point
36	Zip code
10	Non-standard Polygon

For parsed records with a raw spatial scale smaller than the county, such as points, we aggregate results to the county. There is no standardization of the significance of point representations of outages. For example, one point on an outage map could indicate a substation, a centroid of an area experiencing outages, or it could be one meter without power. Aggregating point level outages to the county level resolves some of the potential conflicting interpretations of this data. We assume point data represents outages exclusively within the containing county. This could induce errors if the point represents the centroid of an affected area that in fact crosses county boundaries.

For parsed records which cross county borders (i.e., some zip codes and polygons), we proportionally allocate customers to the intersecting counties based on the share of polygon area in each county. Outages reported by polygon have varying levels of granularity, as these polygons are not consistent across outage maps. For example, outages reported in a polygon could indicate a neighborhood block all the way up to the service territory of the utility itself. If outages are reported in a polygon that represents the service territory, we collect this data only if the entire utility falls within the boundaries of one county. Polygons that cross county lines have outages divided into counties based on proportion of geometry overlap. Because of the coarseness of this approach that does not currently consider population, we collect outage data by county and by point whenever possible.

### Collection errors

The EAGLE-I system records the type of collection errors encountered by each parser with each 15-minute run. The web parser system has scraped approximately 400 websites every 15 minutes for 8 years. For the most recent five years, we have recorded the types of errors that are commonly encountered with our collection process, in the context of over 70 million unique visits (Table 3). Parser errors and connection errors are the most common, as these are the error types commonly flagged when a map URL is no longer operating as expected. Since these error types will be flagged within 15 minutes of detecting a change in the map’s URL behaviour, we are notified in near real-time of the need to update our outage data source to ensure we minimize the gap in collected outages. Invalid location errors are the least consequential, as this typically indicates a county or state should be added to the coverage or when utilities report county names differently than the EAGLE-I designation, as an outage was reported in a geographic area not currently linked to the utility. We still collect and record all outage information for all counties currently linked to that utility, but we are notified of a new area that we can now add to the coverage. This is typically seen when a county expands their coverage and adds a new customer outside of their typical service territory range, and this error helps us stay up to date on service territory changes in between EIA-861 annual updates. This error can also occur if a utility outage map reports outages with invalid coordinates, such as an accidental swap of the latitude and longitude values. A timeout error is typically seen when an outage map has a high number of outages or a high amount of traffic, such as during an extreme weather event. When this occurs, our collection process will hit the maximum time allowed waiting for an individual map to load and will move on to the next utility map to avoid hanging up our ETL process on one map. Outages not recorded due to a timeout error are typically captured in the next 15 minute interval, when the parser comes back to that website. The collection error types in which we do not collect any outages for that run for that utility are parser, connection, timeout, runtime, cache, and scheduler. The error types that still collect some outages but miss partial data are location, sub-utility, and webdriver errors.

**Table 3:** The total number of each collection error type ranked by frequency encountered from 2017–2022, across all utility parsers.

Error Count	Parent Type	Specific Type
**500,026**	Scraper	PARSER_ERROR
**268,487**	Connection	CONNECTION_ERROR
**133,409**	Unknown	UNKNOWN_ERROR
**117,523**	Location	INVALID_LOCATION
**60,001**	System Failure	TIMEOUT
**13,074**	Sub-Utility	INVALID_SUBUTILITY
**1,370**	Database	CACHE_ERROR
**1,122**	System Failure	RUNTIME_ERROR
**160**	Scraper	WEBDRIVER_ERROR
**2**	System Failure	SCHEDULER_ERROR

These errors occur in the context of over 70 million unique visits from our web-parsers to utility websites.

### Near real-time error correction

The EAGLE-I platform has an active user base of state and federal emergency responders and other government agencies. These users frequently make use of the platforms “bug report” feature to point out issues such as a map URL temporarily out of order, producing a gap in coverage. Similarly, utilities occasionally display incorrect or out of date outage estimates, sometimes maintaining a record of outages after they have been resolved on the ground. When these issues are reported, we correct the dataset in near real time based on the best available information. The outage values used to correct these errors come from diverse sources, potentially including first or second-hand reports from emergency responders, or provided by the utility itself in an alternative format such as social media posts when their map URL is not functioning. In the data provided here, these near real time corrections are included without identification. EAGLE-I also enables select privileged users, such as FEMA Regional Coordinators, to manually overwrite parsed data based on their local situational awareness and expertise. This is most typically used in the case of large outage events, such as in response to hurricanes or wildfires. Finally, we actively seek feedback from our user base. Our user base is frequently interested in using the historic outages provided by EAGLE-I to evaluate a past event response, understand the typical distribution of outages in their area based on certain event types, and similar types of analysis. When these users notice gaps, sharp spikes, or drop offs in outages, we may use this observation to review the historic outages and investigate an error in the parser for that utility.

### Focused coverage expansion

The percentage of US electric customers for which EAGLE-I reports outage data has increased over time, particularly focusing on areas of the country with relatively low representation in the EAGLE-I data. There are many strategies to identify potential outage maps to include. Our primary source of new utility information is form EIA-861, an annual survey of electric utilities. This allows us to identify large utilities in states with low coverage. Using the utility names provided in form EIA-861, we match outage map URL patterns within the Google search engine. Additionally, users send in requests for utilities in their area that they would like to have included in the EAGLE-I outage data, often providing the map URL. Finally, large utilities (over 20,000 customers) not currently included in EAGLE-I coverage are evaluated for potential addition during our annual census of EAGLE-I utilities.

## Data Records

The core of the provided dataset includes eight years of power outage information at the county level from 2014 to 2022 at 15-minute intervals collected from utility’s public outage maps on their websites by the EAGLE-I program at ORNL. Data are available in the Figshare repository at 10.6084/m9.figshare.24237376^22^.

There are twelve data files in this repository: nine outage data files and three supplementary data files. The outage data files follow the naming convention eaglei_outages_YEAR.csv and contain outages for each included calendar year. The file coverage_history.csv includes the modeled customer coverage rate of each state from 2018–2022. The file MCC.csv provides the modeled number of electric customers per county as of 2022. The file DQI.csv presents our Data Quality Index and the four sub-components by year by FEMA Region for 2018–2022.

Below is a complete list of the files included in the dataset, detailing the size of the files and the number of rows:

- eaglei_outages_2014.csv (78.1 MB) (1,689,461 rows)
- eaglei_outages_2015.csv (599 MB) (12,939,158 rows)
- eaglei_outages_2016.csv (619.8 MB) (13,306,025 rows)
- eaglei_outages_2017.csv (698.8 MB) (15,078,365 rows)
- eaglei_outages_2018.csv (999.2 MB) (21,776,807 rows)
- eaglei_outages_2019.csv (1.1 GB) (24,074,123 rows)
- eaglei_outages_2020.csv (1.17 GB) (25,545,518 rows)
- eaglei_outages_2021.csv (1.14 GB) (24,826,103 rows)
- eaglei_outages_2022.csv (1.2 GB) (25,796,466 rows)
- MCC.csv (41 KB) (3,235 rows)
- coverage_history.csv (12 KB) (280 rows)
- DQI.csv (6 KB) (50 rows)

### Outage data

Table 4 provides a small sample of the EAGLE-I outage data. The provided outage data does not include zero outages/customers out. Missing entries are either a customer outage value of zero or a gap in data collection; we do not distinguish between these cases. All data in EAGLE-I is presented in Coordinated Universal Time (UTC). The information contained in each column is defined as follows:

- Fips_code: The FIPS code of the county in which the power outages occurred, for example “12011”
- County: The county name in which the power outages occurred spelled out in text, for example “Broward”
- State: The state in which the power outage occurred, spelled out in full in text format.
- Customers_Out: The total number of customers without power for that county at that timestamp. This number is always an integer. Entries with 0 customers without power are not included in this dataset.
- Run_start_time: Date and timestamp provided in UTC in the format “MM/DD/YY 00:00”. This timestamp marks the beginning of the collection run.

**Table 4:** Example of EAGLE-I Outage Data.

fips_code	county	state	customers_out	run_start_time
01005	Barbour	Alabama	4	1/1/22 0:00
01009	Blount	Alabama	160	1/1/22 0:00
01051	Elmore	Alabama	3	1/1/22 0:00
01055	Etowah	Alabama	4	1/1/22 0:00
01057	Fayette	Alabama	4	1/1/22 0:00
01061	Geneva	Alabama	4	1/1/22 0:00

### Coverage history

Table 5 presents a small sample of the coverage data. Below is a description of all the columns included in the dataset for coverage history:

- Year: The date used to derive the coverage for the given year. For example, the entry “1/1/2019” means all following information in the row is based on the date of Jan 1, 2019.
- State: Postal Service two-character state abbreviation, for example “AL” for Alabama.
- Total Customers: The total number of utility customers in the state on that given date. Customers are derived from the closest EIA-861 customer number updates to the provided date.
- Min_covered: The minimum number of utility customers covered by EAGLE-I in a given calendar year, as reported by EIA-861
- Max_covered: The maximum number of utility customers covered by EAGLE-I in a given calendar year, as reported by EIA-861
- Min_pct_covered: The minimum percentage of coverage for the state seen in a given year
- Max_pct_covered: The maximum percentage of coverage for the state seen in a given year

**Table 5:** Example of coverage history.

year	state	total_customers	min_covered	max_covered	min_pct_covered	max_pct_covered
1/1/18	AK	340543	229424	229424	0.67	0.67
1/1/19	AK	340543	217506	229424	0.64	0.67
1/1/20	AK	328964	158477	224243	0.48	0.68
1/1/21	AK	331443	224243	226079	0.68	0.68
1/1/22	AK	364614	226079	258830	0.62	0.71

Our coverage extent and estimates of total customers changes through time based on reporting to the EIA, as well as changes in how utilities present their outages data in public-facing websites. As a result, coverage gaps occur when a utility changes its name, website, or reporting process. Coverage expansions occur when new utilities are added to the EAGLE-I data collection process.

Increases and decreases in the minimum coverage rate and maximum coverage rate can occur as utilities are dropped from the EAGLE-I data collection process due to changes in their outage reporting, or newly incorporated into the data collection process. In row three of Table 5, for example, we may have stopped collecting data from a single large utility for some period of the calendar year. The maximum customer coverage number in 2020 is the minimum coverage number for 2021, suggesting that the coverage drop was temporary, and rectified within the calendar year.

### Modeled county customers

Table 6 provides a small sample of the modeled county customer dataset. The modeled county customer dataset was created to enable estimations of the percent of customers without power by county, with full methods described in Moehl *et al*.^23^. Below is a description of all the columns included in the dataset for modeled county customers:

- County_FIPS: The FIPS code for each county for which we have modelled county customers. If a FIPS code is missing from this list, we are missing information in EAGLE-I for utilities that have customers in this county.
- Customers: The modelled result for the number of electric utility customers living in this county

**Table 6:** Example MCC Data.

County_FIPS	Customers
01001	24619
01003	195253
01005	12400
01007	11037
01009	27074

### Data quality index

The Data Quality Index (DQI) data was created to quantify our best estimate of the comparative quality of our historic outage data. These data include records between 2018 and 2022, at the FEMA region for each year. Below is a description of all the columns included in this file:

- fema: The Federal Emergency Management Agency (FEMA) region.
- year: calendar year
- success_rate: The Success Rate component of the DQI
- percent_enabled: The Percent Enabled component of the DQI
- spatial_precision: The Spatial Precision component of the DQI
- cust_coverage: The Customer Coverage component of the DQI
- max_covered: The maximum number of customers covered in this region/year.
- total_customers: The estimated total number of customers in this region/year
- DQI: the final data quality index, as defined in Eq. 1.

## Technical Validation

In December 2022, EAGLE-I outage data covers 456 utilities and conglomerates. We assess data accuracy, comprehensiveness, and quality using three main strategies including:

1. We assess the comprehensiveness of these data in comparison to the entire population of US utilities by comparing our utility meta-data to the U.S. Energy Information Administration’s Annual Electric Power Industry Report with each annual EIA-861 release^18^.
2. We ensure that our collected data are consistent with our listed population of included utilities by performing an annual census of included utilities to catch silent failures or errors in the data parsing process.
3. Finally, we estimate the external validity of our collected data through a normalized data quality index for each utility. Our annual census and cross-checking with EIA-861 releases serve as our core verification strategy, ensuring that the data reported is as consistent as possible with what is publicly known about utility location, customer population, and outage histories. The data quality index serves our core validation strategy.

**Fig. 2:**
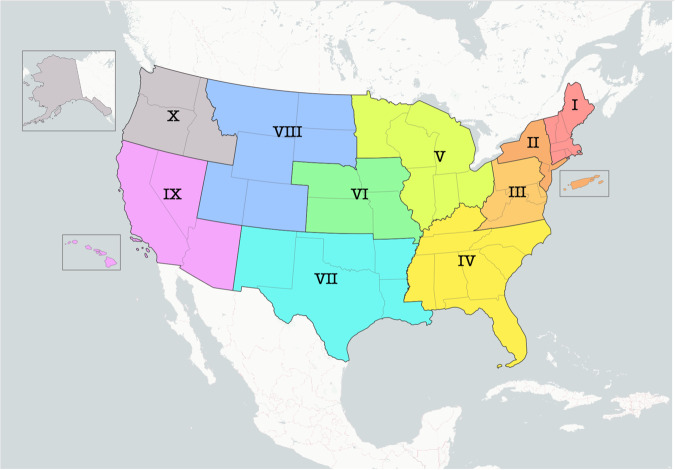
United States FEMA Regions.

Figure 2 presents FEMA regions across the US. We have aggregated our data quality metrics to the FEMA region in this paper for two reasons: (1) to avoid releasing data quality information about individual utilities and (2) a large portion of the EAGLE-I user base works with FEMA regions frequently, so this is a common way that we view our data.

### Data comprehensiveness

The U.S. Energy Information Administration (EIA) produces an Annual Electric Power Industry Report (form EIA-861). This report describes the number of electric utility customers for each US utility and the counties served by each utility. We assess our records of covered utilities against each annual EIA-861 release for two key pieces of information: changes in customer populations and changes in the utility-county relationship. In both cases, we identify inconsistencies between our utility data and that reported by the EIA and assess the most likely real-world condition.

The most critical use of form EIA-861 is to maintain up-to-date measures of customer populations. These are used to calculate outage percentages by state or utility, evaluate our coverage rates nationally and by state, and assess the plausibility of reported outages. We annually update utility, state, and our modelled county level customer populations based on form EIA-861. We assess large or unexpected changes in customer populations on a case-by-case basis. In some instances, we find a utility is no longer covered by the EIA-861 data but in fact still exists and is still reporting outages. This update also flags new utilities (large increases in coverage), utilities that have dissolved (large decreases in coverage), and other potential errors in our records of state coverage or customer populations.

Changes in the utility-county relationship occur when new utilities are created, existing utilities expand their service area, or when utilities close, merge, or contract their service area. When new utilities are added to form EIA-861, we update our state level customer estimates to maintain accurate representation of our coverage rate. We also assess the feasibility of including the new utility in our population of covered utilities. When utilities expand their coverage area, there can be a lag between when we observe outages in a new geography, and when the expanded service area is included in an EIA report. This means that we sometimes observe service area expansions well before this information is included in form EIA-861. We review all discrepancies between EIA county and state coverage with our records of past outages, and if the utility has collected outages from an area within the past year, we continue to include it in our coverage list for that utility. Utility name changes are a good signifier of mergers. When we identify a utility merger, we review the utility information from public data to ensure customers, URL information, name, and all metadata are updated to reflect this merger. Utility mergers are most often identified through our comparison of significant changes in utility names between sequential EIA-861 releases.

When a utility is reported as closing, merging, or reducing its’ service area in EIA-861, we manually determine whether this reduction in scope appears consistent with the utility’s self-reported outage information. We check historic outages for the utility within the past year. If recent outages are found, no changes are made to the database, and the county remains included in the utility’s service area. However, if no recent outages are recorded, we examine the utility’s website. Through coverage territory maps, lists of covered areas, or the outline of the outage map territory, we assess whether the area is indeed no longer served by the utility. In rare cases, utilities maintain operation but stop reporting data to the EIA. We identify these cases by comparing year over year EIA-861 forms. Upon examination of utility websites for those which drop out of the EIA-861 data, if we find no evidence that it has merged and do find evidence that it is still operating, we track the utility through manual assessments of its website and other public information until it is included in EIA-861 again.

This process is crucial to strike a balance between accurately representing the areas served by each utility and avoiding the inclusion of counties that EAGLE-I does not cover. It is imperative not to collect outages from states or counties not listed as covered by a utility in the database. Any location errors flagged by the parser responsible for collecting outage information can be rectified, but during this change, there is a risk of missing out on recording outages. Conversely, if coverage areas are incorrectly included, the accuracy of outage percentages may be compromised, misrepresenting the severity of outages when the total customer denominator is inaccurate.

### Annual census of EAGLE-I utilities for verification

In advance of the North Atlantic hurricane season, we perform an annual census of utilities included in the EAGLE-I data outage records with the goal of identifying silent failures and errors. There are many ways we might incorrectly collect data from a utility and not immediately notice; this workflow ensures that each utility is thoroughly examined at least annually. This labour-intensive process typically takes two months.

In the annual census, for each utility, we ensure that:

1. The outage map URL on record is the most up-to-date outage map available.
2. Recent outages are being reported on our interface. At the beginning of the census process, if a utility has not had an outage within the most recent month, we monitor the utility for another month or until an outage occurs. If two months pass with no outages, we assess whether the outage map is no longer updating, the parser has failed, or some other failure has occurred.
3. We are collecting the finest spatial resolution data available. If we identify a source with higher resolution data (for example from county to point), we change the parser to incorporate that higher resolution data.
4. The outage numbers reported by our parser are consistent with the outage map on the utility’s website.
5. Our collection process accurately divides and assigns outages to each county.
6. Coverage territory for each utility matches the area in the outage map.
7. Recent reported outage histories are realistic. For example, if we observe long-term recurring outages, or wild discontinuities in outage numbers over the course of a few hours, we investigate the outage map and parser to identify and correct any potential errors.

When gaps, errors, or inconsistencies are identified in the outage data during the annual census, we correct them – going forward- to the greatest extent possible. For each of the seven validation steps encompassed in the annual census, if errors are discovered they are corrected in the data going forward. In step one, outage maps are updated to the most update outage map available. Step two assures that outage maps are genuinely updating and takes corrective action if one is not. Step three increases the spatial resolution of our data, when it becomes available. Step four verifies that our code is reproducing the utility reported data correctly. Steps five and six ensure that outages are allocated to the appropriate counties. Step seven assesses reported outages against their real-world plausibility. In this process, we also update utility names and acronyms, and we disable collection for utilities that are no longer operating. Overall, this annual census ensures that our records of historic outages are consistent with that reported by utilities and represent plausible outage events.

### Data quality index

We produce a Data Quality Index (DQI) which synthesizes four key characteristics of the reported outage data:

1. Success Rate (*S*): The customer-weighted share of utility-level collection periods for which our parsers finished without error in each year.
2. Percent Enabled (*E*): The customer-weighted share of utility-level collection periods parser was enabled and seeking data in each year.
3. Customer Coverage (*C*): Total number of customers covered by EAGLE-I captured utilities, over total number of customers in the FEMA region as reported by EIA in each year.
4. Spatial Precision (*P*): Qualitative index of input data precision, bounded from 0 (lowest precision) to 100 (highest precision). Data reported at the county level receives a score of 100, point information a score of 75, zip code level data receives a score of 50, and any non-standard polygon receives a score of 25.

DQI components S, E, and P are recorded at the utility level. The annual FEMA region measure for each component is the customer-weighted average of utility-level data. DQI component 3 (customer coverage) is directly calculated as a rate for the FEMA region. Thus, *X*_*it*_, standing in for *S*_*it*_, *E*_*it*_ and *P*_*it*_ is estimated as:

1 $${X}_{it}=\frac{{\sum }_{u\in i}{X}_{ut}{w}_{ut}}{{W}_{it}}$$

Where *Xut* is the data quality component for each utility in year *t*, *w*_*ut*_ is the number of customers per utility per year, and W_it_ is the total number of customers in FEMA region i in year *t*.

**Fig. 3:**
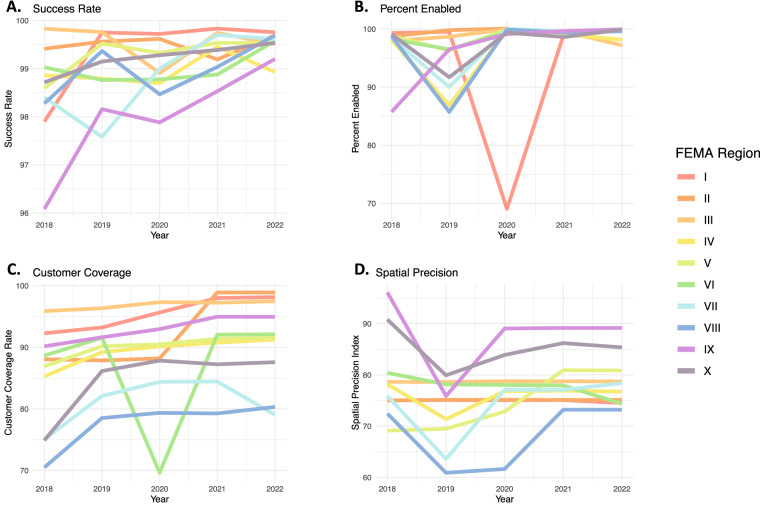
The four sub-components of the Data Quality Index, displayed by FEMA Region and year. Note the four y-axes do not share the same limits, although each metric is bounded between 0 and 100 by definition. Meaningful increases in the success rate and customer coverage metrics contribute to the overall improvements in Data Quality during the study period.

Figure 3 shows the annual customer weighted average for each component for each FEMA region by year. The FEMA region numbering scheme begins with I in the Northeast, and generally moves from North to South and East to West. Longer wavelength colours (reds and oranges) are in the US east, and short wavelength colours (blue and violet) are in the western US.

The DQI is a weighted sum of each of the four DQI components after they have been rescaled to range from 0 to 100:

2 $$DQ{I}_{it}=0.4{S}_{it}+0.3{E}_{it}+0.2{C}_{it}+0.1{P}_{it}$$

where *i* indexes the 10 FEMA regions and *t* indexes the year. The four components have unequal weights because the Success Rate and Percent Enabled metrics are viewed as being the most significant indicators of how well the data represents real world outage conditions.

Unfortunately, we lack formal records of data quality between 2014 and 2017, so the DQI is produced from 2018–2022 only.

These data indicate the overall increase in data quality during the assessed period. Figure 3A shows a consistent increase in the share of parser runs that complete without error through time, for every FEMA Region. In Fig. 3B, the significant drop in Percent Enabled in 2020 for FEMA Region I is due to a change in how outage data were reported. One large utility was subsumed into an even broader utility aggregator, leaving both utilities showing partial year coverage, even though the customers were covered by *some* utilities for greater than 99% of the year. We lack a systematic way to track these transfers of coverage, so coverage expansion and transfers can be associated with decreases in the percent enabled metric.

Figure 3C shows the estimated percent customer coverage seen each year aggregated to the FEMA region from 2018–2022. Utilities can be added or dropped from EAGLE-I coverage any time in the year; we present the annual maximum customer coverage rate. In 2022, FEMA Regions I, II, and III each have coverage rates above 97%, while regions IV, V, VI, and IX have coverage between 90 and 95%. All FEMA regions show increasing coverage rates over time. We see a 10-percentage point jump in FEMA region II with the addition of Puerto Rico in 2021. FEMA Region VIII jumped from 70% Coverage in 2018 to 80% Coverage in 2022. The one year drop in coverage rates in FEMA region VI is due to changes in estimated total customer population in Texas.

The 2019 declines in spatial precision metrics shown in Fig. 3D are associated with a handful of large utilities that changed their reported data from county to a non-standard polygon.

**Fig. 4:**
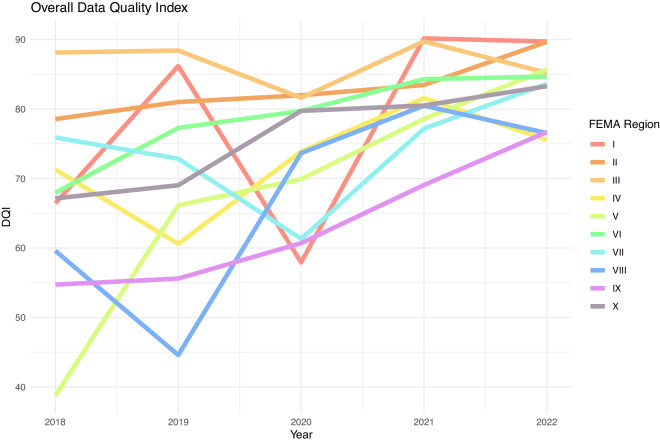
Overall Data Quality Index (DQI). The DQI is comprised of a weighted mean of four data subcomponents, and estimated at the by FEMA Region and year.

Figure 4 presents the full Data Quality Index results. We can see significant improvements in overall data quality. This is driven by increases in each metric, but most significantly in the Success Rate metric.

## Data Availability

The code used to curate the EAGLE-I data for this article is available within the Figshare data repository: 10.6084/m9.figshare.24237376^22^.
